# Skeletal Muscle Remodeling in Response to Eccentric vs. Concentric Loading: Morphological, Molecular, and Metabolic Adaptations

**DOI:** 10.3389/fphys.2017.00447

**Published:** 2017-07-04

**Authors:** Martino V. Franchi, Neil D. Reeves, Marco V. Narici

**Affiliations:** ^1^MRC-ARUK Centre for Musculoskeletal Ageing Research, School of Medicine, University of NottinghamDerby, United Kingdom; ^2^Laboratory for Muscle Plasticity, Department of Orthopaedics, Balgrist University Hospital, University of ZurichZürich, Switzerland; ^3^Faculty of Science and Engineering, School of Healthcare Science, Manchester Metropolitan UniversityManchester, United Kingdom

**Keywords:** eccentric exercise, concentric exercise, eccentric contraction, muscle architecture, muscle remodeling, muscle hypertrophy, muscle signaling, mechanotransduction

## Abstract

Skeletal muscle contracts either by shortening or lengthening (concentrically or eccentrically, respectively); however, the two contractions substantially differ from one another in terms of mechanisms of force generation, maximum force production and energy cost. It is generally known that eccentric actions generate greater force than isometric and concentric contractions and at a lower metabolic cost. Hence, by virtue of the greater mechanical loading involved in active lengthening, eccentric resistance training (ECC RT) is assumed to produce greater hypertrophy than concentric resistance training (CON RT). Nonetheless, prevalence of either ECC RT or CON RT in inducing gains in muscle mass is still an open issue, with some studies reporting greater hypertrophy with eccentric, some with concentric and some with similar hypertrophy within both training modes. Recent observations suggest that such hypertrophic responses to lengthening vs. shortening contractions are achieved by different adaptations in muscle architecture. Whilst the changes in muscle protein synthesis in response to acute and chronic concentric and eccentric exercise bouts seem very similar, the molecular mechanisms regulating the myogenic adaptations to the two distinct loading stimuli are still incompletely understood.

Thus, the present review aims to, (a) critically discuss the literature on the contribution of eccentric vs. concentric loading to muscular hypertrophy and structural remodeling, and, (b) clarify the molecular mechanisms that may regulate such adaptations.

We conclude that, when matched for either maximum load or work, similar increase in muscle size is found between ECC and CON RT. However, such hypertrophic changes appear to be achieved through distinct structural adaptations, which may be regulated by different myogenic and molecular responses observed between lengthening and shortening contractions.

## Lengthening vs. shortening contractions

It is known that skeletal muscle can contract either by shortening or lengthening (*concentrically* or *eccentrically*, respectively). During concentric contractions the muscle shortens and exerts a force, which is transmitted via the tendon to the joint, enables movement to occur and causes a change in joint angle. Eccentric contractions occur in everyday motor activities and usually are responsible for two important characteristics in natural locomotion. Eccentric contractions allow the dissipation of mechanical energy during body deceleration (Konow and Roberts, 2015; e.g., descending stairs/walking downhill, in which the quadriceps and plantar flexors muscles generate force while lengthening, to exert a breaking action against downward movement and to maintain balance), but they also allow the conversion of kinetic energy into elastic energy of tendons (Hoppeler, 2014). Such energy is then regained during limb support, resulting in less muscle work and energy required in locomotion.

Eccentric and concentric contractions fundamentally differ one from the other from a mechanical, metabolic and neural control point of view. Moreover, recent evidence obtained in our laboratory shows that distinct differences in terms of muscle morphological adaptations to resistive training exist between eccentric and concentric contractions.

### Eccentric contractions can produce greater force than concentric contractions through different mechanisms of force generation

**Figure 1:**
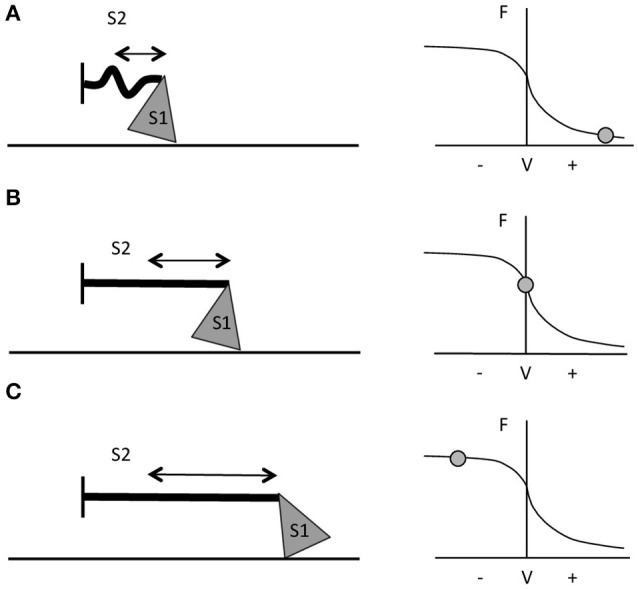
Schematic representation of myosin S1 and S2 segments behavior during different contractions and at different respective portions of the F-V curve. **(A)** During fast shortening muscle actions, S2 complex will not fully stretch, hence myosin will apply lower pulling force onto actin (i.e., the thinner straight line in the figure). **(B)** During slower shortening contractions, up to when the shortening velocity would be equal to 0 (i.e., isometric contractions), the S2 segment will be fully stretched and therefore myosin will be able to apply greater pulling force onto the thin filament. **(C)** During lengthening actions, myosin S2 complex will be able to stretch even further (figure adapted from Jones et al., 2004).

The two contraction types involve different mechanisms of force generation at the contractile protein level; this constitutes one of the main reasons for the greater force production during active lengthening compared to shortening. Muscle force development is the result of the interaction between the contractile filaments. Maximum force is produced when the overlap of the myosin and actin filaments enables the formation of the maximum number of cross-bridges, which occurs at the optimum sarcomere length (Gordon et al., 1966; Huxley and Simmons, 1971). The force developed by a muscle not only depends on sarcomere length and cross-bridges formation, but also on the velocity of shortening (Hill, 1938) or lengthening (Katz, 1939). During shortening contractions *in vitro*, the force generated is always lower than in isometric contractions (for the same level of muscle activation). This occurs because, the quicker the movement, the lesser the number of cross-bridges formed (Huxley, 1957) and higher rate of cross-bridges detachment (Rome et al., 1999). The greater the velocity of contraction, the shorter the time in which myosin can bind to actin. Moreover, during fast movements, the S2 complex of myosin molecule (i.e., the flexible fragment of the myosin tail close to the globular head—Rayment et al., 1993; VanBuren et al., 1994) will not be fully extended, resulting in compression of the S2 complex and in a lower pulling force applied by the thick filament on actin (Figure 1A). When velocity of movement approaches 0, then, not only will a larger number of cross-bridges be attached but also myosin S2 complexes will be fully stretched and able to pull onto the actin filaments to produce bigger values of force (Figure 1B; Huxley and Simmons, 1971; Jones et al., 2004).

*In vitro*, with the increase of lengthening velocity, the force developed rises until it reaches a plateau at a value close to 1.8 times the maximum isometric force (Katz, 1939; Lombardi and Piazzesi, 1990). *In vivo*, the eccentric muscle force is lower than the eccentric force obtained *in vitro* (about 1.2 times the maximum isometric force) and this is probably due to neural inhibition (Amiridis et al., 1996; Babault et al., 2001; Beltman et al., 2004); nevertheless, it is still greater than the one generated by either isometric or concentric contractions (Westing et al., 1988; Aagaard et al., 2000). According to Huxley's model, the greater force produced during active lengthening could be due to a further greater stretch of S2 portions of myosin, occurring first at slow velocity of stretching (Figure 1C). As the lengthening becomes faster, fewer myosin heads will be able to bind to actin, but a good number of these will just remain in the attached position; if these cross bridges are stretched even more, they will inevitably be forced to detach. The proposed mechanism is that these thick filament heads would be able to re-bind to the thin filament very quickly, which maybe representing the reason why muscles are able to develop high forces during lengthening contractions and at lower energetic cost (Lombardi and Piazzesi, 1990; Jones et al., 2004).

The observation that the force generated during active muscle lengthening is substantially greater than the values obtained during isometric and shortening contractions suggests a potential contribution of some structural scaffold to the force developed (as myofibrils would be forcibly lengthened beyond actin-myosin optimal overlap zone; Herzog et al., 2008; Herzog, 2014). The work by Nishikawa (Nishikawa et al., 2012) suggests that the giant protein titin could be involved in muscle contraction mechanics, acting as an internal spring able to store and release elastic potential energy. It has been demonstrated that, during the cross-bridges cycle, actin rotates as myosin translates (Morgan, 1977). The theory proposes titin behaving as a “winding filament,” which is activated by Ca^++^ release and winds upon the actin filament when the latter is rotated by myosin translation in cross-bridges (Monroy et al., 2012); thus, titin will “actively” participate to the generation of force of a muscle through its stiffening when winded up onto actin during active lengthening contractions (please see Hessel et al., 2017 for further illustrations).

### Eccentric contractions are more efficient than concentric contractions

Fundamental differences exist between eccentric and concentric contractions in terms of energy cost. Early observations by Bigland-Ritchie and Woods (Bigland-Ritchie and Woods, 1976) showed that for the same movement (cycling) speed, the energy cost of positive work (shortening) is about six-fold greater than that of negative work (lengthening). Thus, eccentric contractions have been advocated as particularly suitable for recovering muscle mass and strength in elderly and clinical populations (Hoppeler, 2014; Mitchell et al., 2017). The features of metabolic and energetic costs of ECC compared to CON contractions have been recently reviewed by Professor Hoppeler (Hoppeler, 2016).

### Eccentric contractions present different neural control strategies compared to concentric contractions

Concentric and eccentric contractions seem to considerably differ also in terms of neural drive (Duchateau and Enoka, 2008). EMG amplitude is typically greater during shortening than during lengthening contractions (Tesch et al., 1990; Westing et al., 1991; Amiridis et al., 1996; Kellis and Baltzopoulos, 1998; Aagaard et al., 2000; Komi et al., 2000) and voluntary activation can be lower during lengthening contractions (Amiridis et al., 1996; Babault et al., 2001; Beltman et al., 2004). During maximal voluntary contractions, it seems well established that there is a deficit in voluntary activation in eccentric contractions. Indeed, due to the greater force capacity of muscle during lengthening contractions, fewer motor units are recruited and the discharge rate is lower during lengthening contractions compared with shortening contractions (Duchateau and Enoka, 2016). However, the mechanisms responsible for the activation deficit of eccentric contractions are only partly understood. While some authors argue that this inhibition may originate from excessive tension applied to the tendon complex due to excitation of the Golgi tendon organ, leading to reduced motor neurone responsiveness to incoming descending inputs (Westing et al., 1991; Aagaard et al., 2000); a decline in output from the motor cortex or an increase in presynaptic inhibition of facilitation from the periphery are also likely to be involved (Duchateau and Enoka, 2016).

## The contribution of chronic concentric vs. eccentric loading to muscle hypertrophy

ECC resistance training, when performed at high intensities, is associated with greater increases in both total and eccentric strength compared to CON RT (Roig et al., 2009; Isner-Horobeti et al., 2013; Baroni et al., 2015). However, while a substantial wealth of literature exists on hypertrophic responses to concentric and eccentric training (Jones and Rutherford, 1987; Higbie et al., 1996; Seger et al., 1998; Blazevich et al., 2007; Reeves et al., 2009; Moore et al., 2012), the mechanisms regulating these changes have not yet been fully elucidated, especially in humans.

There is still some confusion regarding the singular contribution of both contractions to muscular hypertrophy. Lengthening contractions have the potential to generate greater muscle force than isometric and shortening ones (Katz, 1939; Westing et al., 1991; Cook and McDonagh, 1995); for this reason (i.e., the possibility to train with greater loads), the general consensus is that eccentric exercise may have the potential to promote larger increases in muscle size and strength compared to concentric and isometric training (Roig et al., 2009). However, other authors (Wernbom et al., 2007; Hyldahl and Hubal, 2013) have suggested that if the two types of loading are performed at same intensity and/or work volume, then it is difficult to establish which is the best training mode, as significant hypertrophy is reached in either case. The most relevant studies that compared ECC vs. CON training and the correspondent increase in muscle size are presented in the following subsections.

The investigations that have been included in this table are those that compared eccentric only vs. concentric only loading paradigms within a young population. Many investigations differed one from the other on what has been considered/chosen as index of muscle hypertrophic adaptations to RT. Therefore, studies have been grouped in 6 different categories, depending on the different index (and thus method of assessment) of hypertrophy: muscle girth (Table 1.1), muscle anatomical cross-sectional area (ACSA; obtained from either CT or MRI scans) (Table 1.2), muscle thickness (obtained from ultrasound scans; Table 1.3), muscle volume (obtained from MRI scans; Table 1.4), fiber type II CSA (obtained from muscle biopsies and microscope analysis; Table 1.5), and thigh muscle fat free mass/lean mass (obtained by region of interest analyses from DXA; Table 1.6).

**Table 1.1:** Studies investigating ECC only vs. CON only RT and the contribution to the increase in muscle girth within a young healthy population.

**Studies**	**Population & muscle group**	**Intensity/duration**	**Matching**	**Results**	**Interpretation**
Komi and Buskirk, 1972	Young—Elbow Flexors	MVC—7 Wks	No	ECC only increase	Muscle Girth: ECC> CONC in 2 studies (elbow flexors, pectoralis major). Similar responses in 2 out of 4 studies using Isokinetic or Isotonic loading
Duncan et al., 1989	Young men—Quadriceps	MVC—6 Wks	No	No sig changes for ECC and CON	
Ben-Sira et al., 1995	Young women—Quadriceps	65% 1RM CON—10 Wks	No	Similar ECC and CON increase	
Coratella and Schena, 2016 (^*^)	Young men—Pectoralis Major	CON = 85% 1RM, ECC = 120% CON 1RM—6 wks	Matched for load and training volume	ECC only increase	

(*) *, Investigation performed on already trained subjects*.

**Table 1.2:** Studies investigating ECC only vs. CON only RT and the contribution to the increase in muscle ACSA within a young healthy population.

**Studies**	**Population & muscle group**	**Intensity/duration**	**Matching**	**Results**	**Interpretation**
Jones and Rutherford, 1987	Young men—Quadriceps	80% CON 1RM—12 Wks	No	Similar ECC and CON increase	1- Muscle ACSA ECC> CONC: clear differences in only 2 studies out of 10. 2- Similar responses in 8/10 studies using Isokinetic or Isotonic loading. 3- The two loading modalities seem to promote regional hypertrophic responses. 4- When matched for work or load: Similar ECC and CON ACSA increase
Higbie et al., 1996	Young women—Quadriceps	MVC—10 Wks	No	ECC > CON	
Smith and Rutherford, 1995	Young men—Quadriceps	ECC load = 35% grater than CON load	~ Load Match (ECC overload)	Similar ECC and CON increase distal ACSA	
Seger et al., 1998	Young men—Quadriceps	MVC—10 Wks	No	Similar ECC and CON increase ACSA Mid, Distal ACSA: ECC > CON	
Vikne et al., 2006	Young men—Elbow Flexors	MVC—12 Wks	No	ECC only increase	
Blazevich et al., 2007	Young men—Quadriceps	MVC—10 Wks	No	Similar ECC and CON increase	
Moore et al., 2012	Young men—Elbow Flexors	MVC—9 Wks	Total External Work	Similar ECC and CON increase	
Farup et al., 2014	Young men—Quadriceps	%60-80 1RM CON, 120% 1RM CON for ECC group—12 Wks	~ Load Match (ECC overload)	Similar ECC and CON increase	
Rahbek et al., 2014	Young men—Quadriceps	1RM = training load Progressive (please see reference) 12 wks	No	ECC and CON similar increase % ACSA, greater increase in the Whey supplement group vs. control	
Franchi et al., 2014	Young men—Quadriceps	%80 1RM ECC and CON, respective to the training mode—10 Wks	Matched for max relative load and theoretical equivalent neural activation	CON > ECC Mid ACSA ECC > CON distal ACSA Similar proximal ACSA	

**Table 1.3:** Studies investigating ECC only vs. CON only RT and the contribution to the increase in muscle volume within a young healthy population.

**Studies**	**Population & muscle group**	**Intensity/duration**	**Matching**	**Results**	**Interpretation**
Blazevich et al., 2007	Young men—Quadriceps	MVC—10 Wks	No	Similar ECC and CON increase in whole Quadriceps and VL and VM	Muscle Volume: Similar responses between ECC and CON to either Isokinetic or Isotonic RET for whole Quadriceps, VL and VM
Franchi et al., 2014	Young men—Quadriceps	%80 1RM ECC and CON, respective to the training mode—10 Wks	Matched for max relative load and theoretical equivalent neural activation	Similar ECC and CON increase in VL Volume	

**Table 1.4:** Studies investigating ECC only vs. CON only RT and the contribution to the increase in muscle thickness within a young healthy population.

**Studies**	**Population & muscle group**	**Intensity/duration**	**Matching**	**Result**	**Interpretation**
Farthing and Chilibeck, 2003	Young men and women—Elbow Flexors	MVC—8 Wks	No	ECC > CON (at different sites and velocities)	Muscle Thickness: ECC> CONC in only one study (elbow flexors and with different velocities), but similar responses between ECC and CON in 5 out of 6 studies using Isokinetic or Isotonic loading, investigating quadriceps, VL, VM, BFLh and supraspinatus muscles
Blazevich et al., 2007	Young men—Quadriceps	MVC—10 Wks	No	Similar VL and VM ECC and CON increase	
Cadore et al., 2014	Young men—Quadriceps	MVC—6 Wks	No	Similar ECC and CON increase	
Franchi et al., 2015	Young men—Quadriceps	%80 1RM ECC and CON, respective to the training mode—4 Wks	Matched for max relative load and theoretical equivalent neural activation	Similar VL ECC and CON increase	
Timmins et al., 2016a	Young men—Biceps Femoris	MVC—6 Wks	No	No significant changes for ECC and CON	
Kim et al., 2015	Young men and women—Supraspinatus	MVC—8 Wks	No	Similar ECC and CON increase	

**Table 1.5:** Studies investigating ECC only vs. CON only RT and the contribution to the increase in fiber type II CSA within a young healthy population.

**Studies**	**Population & muscle group**	**Intensity/duration**	**Matching**	**Result**	**Interpretation**
Mayhew et al., 1995	Young men and women—Quadriceps	90% maximal CON power—4 Wks	No	CON > ECC	Muscle fiber type II CSA: ECC> CONC in 3 studies out of 5 Similar responses between ECC and CON in 1 study and CON > ECC in 1 study.
Hortobágyi et al., 1996	Young women—Quadriceps	MVC—12 Wks	No	ECC > CON	
Seger et al., 1998	Young men—Quadriceps	MVC—10 Wks	No	No differences between ECC and CON	
Hortobágyi et al., 2000	Young men and women—Quadriceps	MVC—12 Wks	No	ECC > CON	
Vikne et al., 2006	Young—Elbow Flexors	MVC—12 Wks	No	ECC only increase	

**Table 1.6:** Studies investigating ECC only vs. CON only RT and the contribution to the increase in lean mass/fat free mass within a young healthy population.

**Studies**	**Population & muscle group**	**Intensity/duration**	**Matching**	**Results**	**Interpretation**
Nickols-Richardson et al., 2007	Young Women—Quadriceps/Biceps fem—Elbow flexors/extensors	MVC—20 Wks	No	No differences in fat free mass for ECC and CON	Muscle FFM/LM: Similar responses between ECC and CON to either Isokinetic or Isotonic RET for thigh fat free mass and lean mass.
Hawkins et al., 1999	Young women—Quadriceps	MVC—18 Wks	No	Similar ECC and CON increase in Lean Mass	
English et al., 2014	Young men—Quadriceps & Triceps Surae	ECC performed at 0/33/66/100 or 138 % CON load (5 training groups)—8 Wks	Only one ECC group was load matched (138% CON load)	Similar ECC and CON increase in Lean Mass	
Franchi et al., 2015	Young men—Quadriceps	%80 1RM ECC and CON, respective to the training—4 Wks	Matched for max relative load and theoretical equivalent neural activation	Similar ECC and CON increase in Lean Mass	

### Muscle girth

When considering *muscle girth* as an index of muscle hypertrophy in response to ECC vs. CON RT, a total of four studies have been identified. Two of these studies used Isokinetic RT modality (one investigating elbow flexors—Komi and Buskirk, 1972, the other focusing on knee extensors, Duncan et al., 1989), while the other two utilized isotonic RT (one investigating the knee extensors—Ben-Sira et al., 1995–and the other using a bench-press exercise, focusing on pectoralis major girth—Coratella and Schena, 2016). The results are quite conflicting, as only two out of four studies showed superiority of ECC RT. It is noteworthy to mention that these two studies focused on upper body muscles, whereas no significant differences were found between ECC and CON RT in terms of muscle girth increase in knee extensor muscles, irrespective of the modality of exercises (i.e., Isokinetic or Isotonic) adopted. Therefore, it is also important to acknowledge the possible variation that exists among different muscle groups in the responses to similar training modalities.

### Muscle ACSA

When considering *muscle ACSA* as index of muscle hypertrophy in response to ECC vs. CON RT, a total of ten studies have been identified: eight investigated knee extensor muscles while just two focused on the elbow flexors group. Only two studies (Higbie et al., 1996; Vikne et al., 2006- on knee extensors and elbow flexors, respectively) clearly support a superiority of ECC loading in muscle hypertrophy, whereas the other eight investigations presented inconclusive evidence of a superiority of one mode over the other.

**Figure 2:**
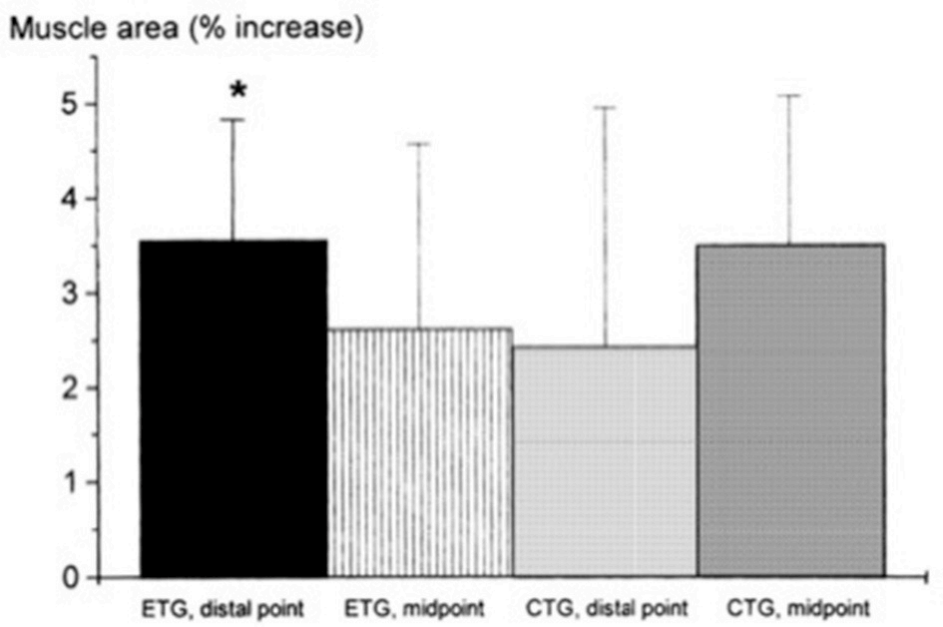
From Seger et al. (1998): preferential distal significant hypertrophy after ECC RT. If the two sites had been considered together as a sum of ACSA across consecutive axial scans, (as in Higbie et al., 1996), the differences between ECC and CON in terms of “whole” hypertrophic (and not regional) response might have shown a different outcome (ECC ~ CON hypertrophy). ^*^P < 0.05.

Seger et al. (1998) reported that ECC RT produced greater increase in ACSA compared to CON RT, but this was only found at the distal portion of the quadriceps while CON RT resulted in greater increase of mid-belly CSA compared to ECC loading (although this difference was not statistically significant, see Figure 2). However, potentially, if the two sites had been considered together as a sum of ACSA across consecutive axial scans, (as in Higbie et al., 1996), the differences between ECC and CON in terms of “whole” hypertrophic (and not regional) response might have shown another outcome.

**Figure 3:**
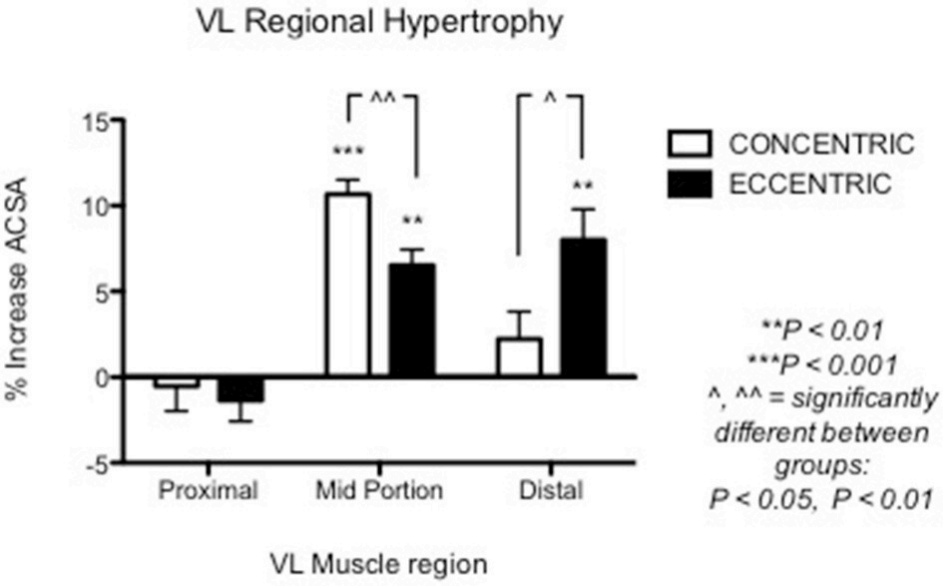
From Franchi et al. (2014): VL Regional hypertophic adaptations to ECC vs. CON RT programs are shown, ECC resulting in greater distal hypertrophy while CON presents larger mid muscle belly increase of CSA.

Nonetheless, other investigations underlined the importance of regional growth (Narici et al., 1989; Smith and Rutherford, 1995; Blazevich et al., 2007). We previously showed that the different contractile stimuli might lead to regional hypertrophy, as presented in Figure 3 (taken from Franchi et al., 2014, where an average of 5 CSA was taken from each vastus lateralis length portion). From this study, it appears that ECC RT induces greater changes in CSA toward the distal portion of the VL muscle, whereas CON RT results in a greater mid-belly associated growth.

In conclusion, the results of the studies that used muscle ACSA as hypertrophy index suggest that there is still not enough evidence for ECC RT to lead to greater muscle growth than CON RT: in addition, when the two training modes are performed while matched either for work or load, changes in muscle size are very similar.

### Muscle volume

When considering *muscle volume* as an index of muscle hypertrophy in response to ECC vs. CON RT, a total of only two studies were found. Neither of the investigations presented significant differences between the loading typologies in the increase of muscle volume after training. It is intriguing to highlight that, in the study from our lab (Franchi et al., 2014), even if different regional morphological patterns of muscle hypertrophy were reported between ECC vs. CON RT (distal vs. mid-belly hypertrophy, respectively), these did not result in significant differences in whole muscle volume. This implies that, if only the peak of ACSA at mid-thigh (scientifically well accepted) would have been used as index of hypertrophy (ECC RT vs. CON RT mid ACSA increase = 7 vs. 11%), the study in question would have potentially provided slight different outcomes than the ones reported (ECC RT vs. CON RT whole volume increase = 6 vs. 8%).

### Muscle thickness

When considering *muscle thickness* as index of muscle hypertrophy in response to ECC vs. CON RT, a total of six studies were identified. Five out of the six examined studies failed in finding significant differences between loading paradigms on knee extensors, biceps femoris long head or supraspinatus muscles. One study (Farthing and Chilibeck, 2003) showed that, for the elbow flexors, at the mid and distal site, high velocity ECC RT resulted in higher increase of MT compared to high and slow velocity CON RT. However, when slow velocity ECC RT was compared to slow velocity CON RT, no differences were found in MT change between exercise programs. Nonetheless, at the proximal site, the study showed a substantial superiority of High Velocity ECC RT in producing greater changes in MT.

In conclusion, the results of the studies that used MT increase as hypertrophy index suggest that no clear differences could be found between ECC and CON RT.

### Muscle fiber type II CSA

When considering *muscle fiber type II CSA* as index of muscle hypertrophy in response to ECC vs. CON RT, a total of five studies were considered. Data on type II muscle fibers only have been reported as it has been showed the preferential recruitment (and consequent hypertrophy) of these fibers in eccentric actions (Fridén et al., 1983; Hortobágyi et al., 1996). Indeed, it appears that ECC loading may also favor the increase of type II fiber CSA, as three studies out of five showed clear superiority of lengthening contractions as stimulus for such adaptations. Furthermore, there seems to be a close connection between ECC RT, performed at higher intensities/velocities of movement, and fiber type II increase in CSA (and percentage too). This connection was the object of previous works, which investigated the effect of ECC training, performed in isolated manner (Paddon-Jones et al., 2001; Shepstone et al., 2005) or as overload phase in mixed-model designs (Hather et al., 1991; Hortobágyi et al., 2000; Friedmann-Bette et al., 2010). All studies found a significant increase in either of CSA or distribution of type II fibers in human skeletal muscle in response to ECC RT. Conversely, just one study (Mayhew et al., 1995) have observed greater fiber type II CSA change after CON compared to ECC RT (both contractions performed at 90% of Max CON power).

### Muscle lean mass/fat free mass

When considering *muscle lean mass/fat free mass* as index of muscle hypertrophy in response to ECC vs. CON RT, a total of four studies were found. None of the investigations presented significant differences between the loading typologies in the increase of muscle volume after training.

### Mixed concentric/eccentric loading and muscle hypertrophy

While the present review is specifically focused on ECC only vs. CON only RT adaptations, it is important to briefly illustrate the adaptations in muscle hypertrophy in response to modes of RT that use a combination of concentric and eccentric contractions. Previous studies have compared the increase in muscle size (mainly in terms of type I and type II fiber CSA) in response to CON only RT vs. the combination of CON/ECC RT performed with isotonic (Hather et al., 1991) or isokinetic devices (Colliander and Tesch, 1990; Hortobágyi et al., 2000). The results are mixed, as no differences in type I CSA increase were found between loading modes (Colliander and Tesch, 1990) but the combination of CON/ECC RT resulted in greater type II fiber CSA compared to CON only (Hather et al., 1991; Hortobágyi et al., 2000). However, Friedmann-Bette et al. (2010) showed that, when comparing two different CON/ECC RT programs (group 1 = same load displaced between phases vs. group 2 = ECC overload, with ECC load ~1.9 of CON one), only CON/ECC overload RT program resulted in significant fiber type II CSA increase. It therefore appears that, when combining both contraction phases in an exercise regime, the best results for muscle growth are obtained by training paradigms that present some sort of ECC overload. In fact, the highest rate of whole muscle hypertrophy increase in humans is observed after flywheel training (Lundberg et al., 2013; Tesch et al., 2017), which provides accommodate resistance in the full range of motion of the CON phase, with ECC overload (Tesch et al., 2017). Flywheel training has shown to lead to very early hypertrophic and architectural adaptations (visible by MRI-assessed ACSA and ultrasound-derived fascicle length from 21 days after the start of the protocol; Seynnes et al., 2007). Although it is not within the scope of this manuscript to further present mixed CON/ECC loading strategies, it is intriguing to acknowledge that differences exist between muscular adaptations to ECC only or CON only and the combination of both types of contractions (with or without ECC overload) in training regimes.

## The contribution of chronic concentric vs. eccentric loading to changes in muscle architecture

This review aimed to critically discuss the evidence for eccentric and concentric training to lead to different morphological adaptations. Perhaps, a significant difference between the two training modes lies in the contraction-specific regional hypertrophy that has been previously observed (Seger et al., 1998; Franchi et al., 2014; Douglas et al., 2016), where greater eccentric-associated distal growth is juxtaposed to a more pronounced mid-belly hypertrophy after concentric training.

**Figure 4:**
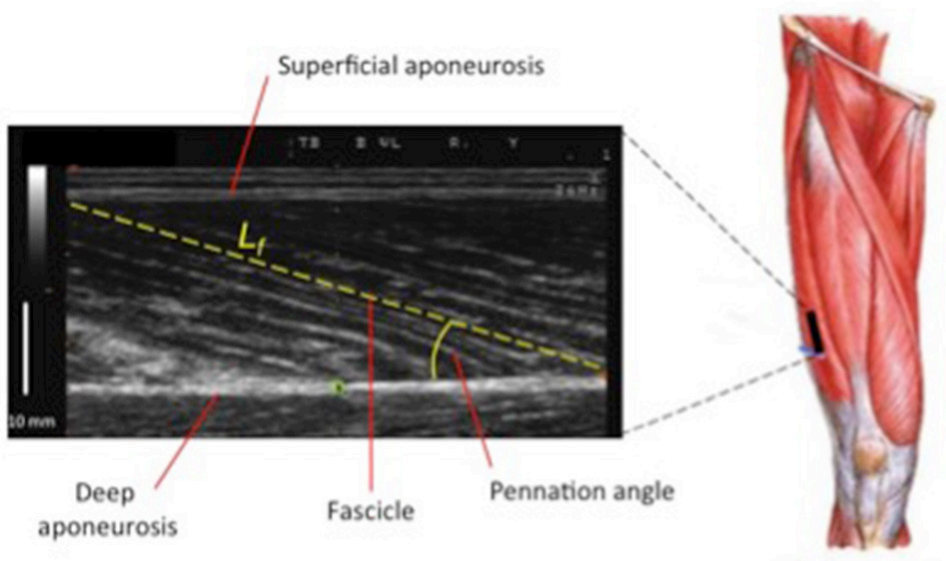
Ultrasound scan of VL muscle: Lf (fascicle length) and PA (pennation angle) represent two of the major features of muscle architecture.

**Figure 5:**
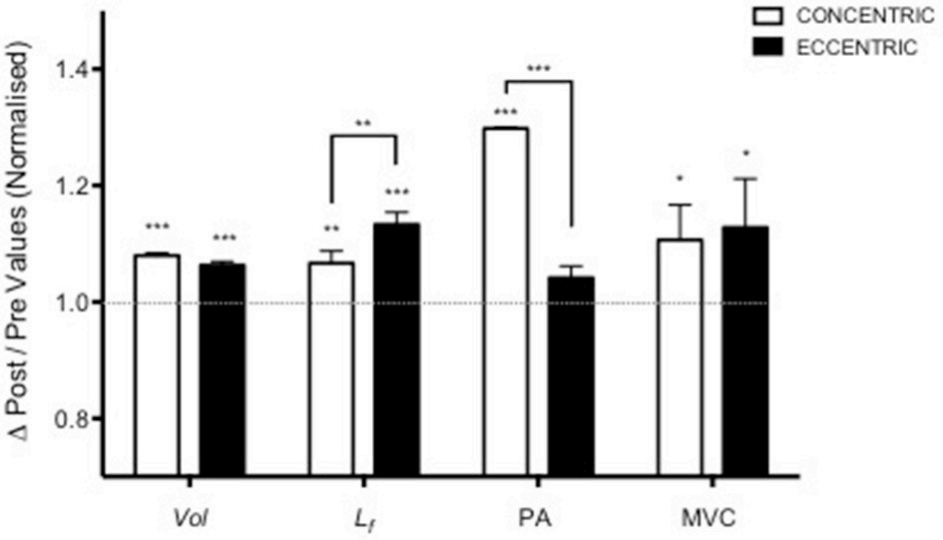
Adapted from Franchi et al. (2014). Contraction-dependent muscle growth in response to eccentric and concentric resistive training in young males. Similar hypertrophy is achieved through two different patterns of structural re-assembly (^*^P < 0.05, ^**^P < 0.001, ^***^P < 0.0001). Y = 1 represent the baseline value, data are normalized to pre-exercise values (Vol, Volume; Lf, fascicle length; PA, pennation angle; MVC, maximum voluntary contraction).

Nonetheless, while similar “whole muscle” growth is often observed as a result of both types of RT, what really seems to differ between ECC and CON loading are the mechanisms of structural remodeling by which the hypertrophic responses are achieved. Our lab has recently shown that different architectural adaptations (measured by using B-mode ultrasound technique, Figure 4) can be found between ECC vs. CON contractions: ECC results in a markedly greater increase in fascicle length (Lf) while CON promotes greater changes in pennation angle (PA), likely reflecting the differential addition of sarcomeres either in series or in parallel, respectively (Reeves et al., 2009; Franchi et al., 2014, 2015) (Figures 5, 7). Therefore, muscle growth is achieved with both loading modalities, but the mechanisms of structural remodeling are contraction-specific (Franchi et al., 2016a). Although, a good number of investigations have focused on architectural responses to ECC RT only, so far, only five studies have compared isolated CON vs. isolated ECC in terms of structural remodeling. Those studies are presented in Table 2.

**Figure 6:**
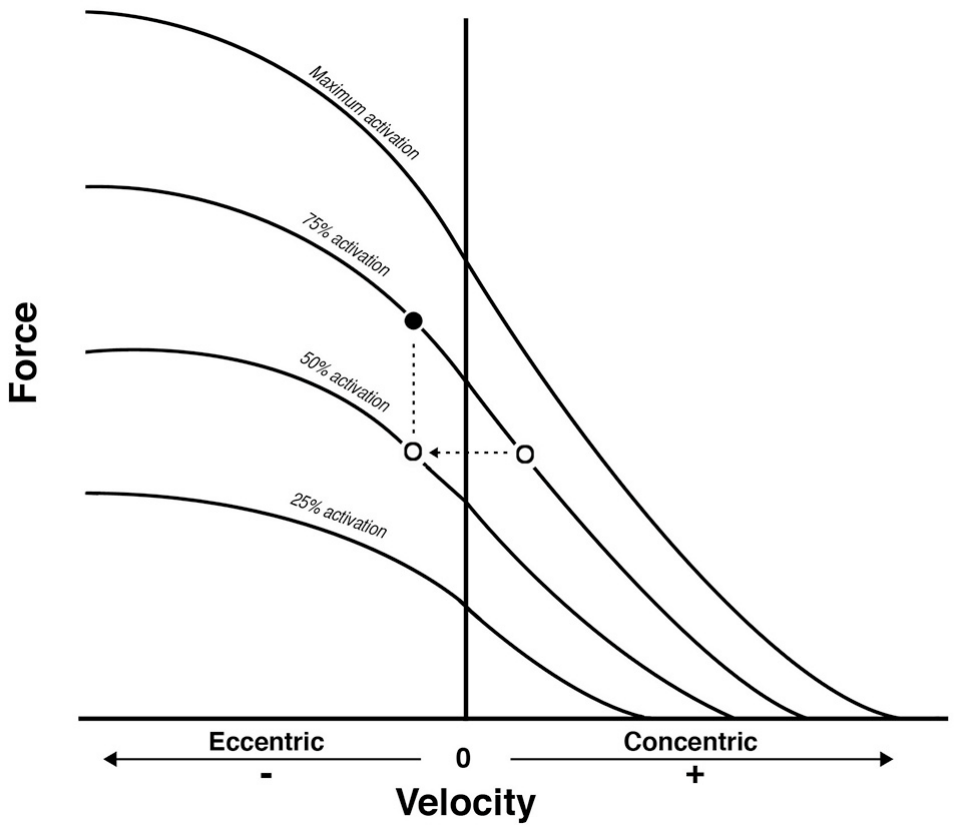
From Reeves et al. (2009): conceptual diagram that illustrates what could theoretically happen when displacing the same external load (empty dots) between concentric and eccentric phases in Conventional RT. As a consequence, the eccentric contraction belongs to a different force-velocity curve of lower neural activation. Because a fundamental requirement of the force-velocity relationship is that all the point of force and velocity should belong to a curve of same neural activation, then the filled dot represent the level of external load that should be adopted to meet such requirement: something that is not occurring in conventional resistance training.

**Figure 7:**
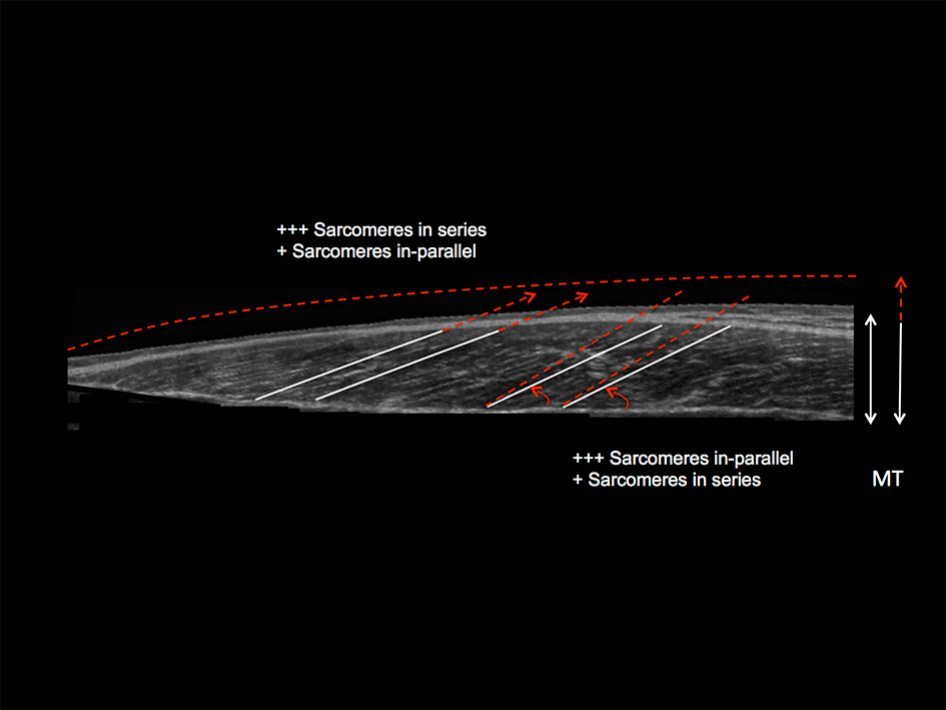
Schematic diagram illustrating the distinct contraction-specific hypertophic patterns in response to chronic ECC vs. CON RT in human vastus lateralis (image acquired by using extended field of view ultrasound technique): a similar increase in MT can indeed be reached either through a preferential addition of sarcomeres in-parallel (usually occurring after CON RT) with an increase in PA, or through a preferential addition of sarcomeres in-series (usually occurring after ECC RT), which is represented by an increase in Lf (Franchi et al., 2014, 2015). +++ = the preferential addition of either sarcomeres in-series or in-parallel (dependent on the contraction mode used) compared to + = likely to happen as a marginal response. The white lines highlight the initial pre-training scenario, whereas the red dotted lines represent a post-training hypertrophic state.

**Table 2:** Summary of studies investigating the architectural adaptations to ECC only vs. CON only loading protocols.

**Studies**	**Population & muscle group**	**Intensity/duration**	**Matching**	**Results**	**Interpretation**
Blazevich et al., 2007	Young men—Quadriceps	MVC—10 Wks	No	Similar Lf, PA and CSA increase in both groups	Muscle Architecture: 4 studies out of 5 show clear distinct architectural adaptations in reponse to either CON or ECC training. CON RET induces a greater increase of PA compared to ECC RET, whereas ECC loading is the predominant stimulus to achieve greater changes in Lf. Only 1 study found ECC and CON to achieve similar architectural remodeling patterns (Blazevich et al., 2007)
Reeves et al., 2009	Older men—Quadriceps	%80 5RM ECC and CON, respective to ECC or Conventional training mode—14 Wks	Matched for max relative load	ECC > CON on Lf increase (20% vs. 8%); CON > ECC on PA increase (35% vs. 5%); Similar increase in MT (~12%)	
Franchi et al., 2014	Young men—Quadriceps	%80 1RM ECC and CON, respective to the training mode—10 Wks	Matched for max relative load and theoretical equivalent neural activation	ECC > CON on Lf increase (12% vs. 5%); CON > ECC on PA increase (30% vs. 5%); Similar increase in VOL (~7%)	
					
Franchi et al., 2015	Young men—Quadriceps	%80 1RM ECC and CON, respective to the training mode—4 Wks	Matched for max relative load and theoretical equivalent neural activation	ECC > CON on Lf increase (5% vs. 2%); CON > ECC on PA increase (7% vs. 3%); Similar increase in MT (~8%)	
Timmins et al., 2016a	Young men—Biceps Femoris	MVC—6 Wks	No	ECC > CON on Lf increase (16% vs.—11%); CON > ECC on PA increase (20% vs.—7%); No significant increase in MT in both groups	

Thus, when considering the results reported in Table 2, the prevalent observation is that the two contractions induce distinct structural adaptations. Since changes in architecture can influence the functional properties of muscles (Narici, 1999; Lieber and Fridén, 2000; Narici et al., 2016), a thorough understanding of skeletal muscle remodeling during hypertrophic states is then fundamental. Conventional (Narici et al., 1996; Alegre et al., 2006), concentric and eccentric loading can result in changes of Lf (Blazevich et al., 2007): however, eccentric-only RT induces greater increase in Lf of the VL muscle than conventional (Reeves et al., 2009) and concentric-only RT (Franchi et al., 2014, 2015). Similar findings have been observed in the biceps femoris muscle (Timmins et al., 2016a). By contrast, Blazevich et al. (2007) found both contractions to induce a similar and significant increase in Lf; however, in this study, the two different loading paradigms were not matched for work, relative load or neural drive. As previously discussed by Reeves et al. (2009) during conventional RT (typically consisting of consecutive concentric and eccentric phases), because the same absolute load is displaced between CON and ECC phases, motor units must be de-recruited to enable lowering of the training load (eccentric phase). Hence in the eccentric phase of conventional RT, muscle activation and relative loading, are considerably lower than those achieved in the concentric phase (Figure 6). Therefore, to guarantee that the ECC component of resistance training would not result under-loaded, Reeves et al. (2009) and Franchi et al. (2014) increased the eccentric training load by about 20% in order to obtain the same level of neural drive (EMG), to meet the fundamental requirement of the force-velocity relation that each value of force and velocity along this curve should belong to the same level of neural activation (Bigland and Lippold, 1954). Such a controlled study design allowed the authors to better compare the two loading modalities.

In relation to the muscle morphological adaptations to CON and ECC training, Blazevich and colleagues suggested that training at greater than normal joint range of motion may explain the similar structural remodeling in response to the two loading modes, instead of the contraction type. This hypothesis seems supported by other studies (McMahon et al., 2014; Guex et al., 2016), which found a greater increase in fascicle length of VL when training at greater ROM of the knee joint. Nonetheless, Noorkõiv et al. (2014) showed that isometric training at longer muscle lengths (i.e., thus almost re-producing an ECC contraction scenario) can induce a substantial increase in Lf. Thus, regarding the findings from Blazevich's study (2007) the authors raise the point of whether more pronounced mechanical stretch might have been applied to single sarcomeres (and thus fascicles) during large ROM CON RT at higher joint knee angle (counting the full leg extension as anatomical zero = 0 degrees) and if this could have affected serial sarcomeres distribution and ultimately the architectural adaptations presented by the aforementioned studies.

There is a substantial bulk of literature showing Lf increases after eccentric-only (Duclay et al., 2009; Potier et al., 2009; Baroni et al., 2013) or isoinertial exercise (Seynnes et al., 2007), reinforcing the concept that muscle longitudinal growth may be intimately related to lengthening contractions. The changes in Lf observed after these loading interventions theoretically imply the addition of sarcomeres in series. It is known from previous animal work that skeletal muscle responds to passive and intermittent stretch by adding new sarcomeres in-series (Williams and Goldspink, 1971; Holly et al., 1980; Goldspink, 1985; Williams et al., 1988; Williams, 1990), a trend observed after exercise regimes/overload (Goldspink, 1999; Proske and Morgan, 2001). Greater addition of serial sarcomeres was found in rats after downhill compared to uphill running (Lynn and Morgan, 1994; Butterfield et al., 2005): thus, it has been suggested that more contractile material placed in series (i.e., reflecting the increase in Lf) can be regarded as a “protective” mechanisms after eccentric exercise induced muscle damage (Morgan and Talbot, 2002), also because of the increase of maximal force produced at longer muscle lengths (Timmins et al., 2016a).

Conversely, an increase of PA has been regarded as a strategy for the muscle to pack more contractile material along the deep tendinous aponeurosis (Gans, 1982; Kawakami et al., 1993): thus, hypertrophy of pennate muscles can be often accompanied by a substantial increase of PA (Ema et al., 2016). However, PA appears to markedly increase after concentric and conventional RT (in which the concentric contraction stimulus is the most dominant, as the intensity is usually based on CON 1RM %), whereas none/small and often non-significant changes are observed in response to ECC loading programmes (Baroni et al., 2015).

Taken together, these results strongly suggest that skeletal muscle is able to respond to different mechanical stimuli by following distinct remodeling patterns, reflecting divergent strategies for the addition of new sarcomeric material in its structural re-assembly (Narici et al., 2016) (Figure 7). This also results in important implications in rehabilitation settings, because different contractions can therefore influence the shift of the optimum length-tension relationship, with notable repercussions on performance and injury prevention (Brughelli and Cronin, 2007; Timmins et al., 2016b). In fact, the addition of serial sarcomeres appears to directly impact the maximum velocity of shortening of muscle fibers (in cat semitendinosus muscle; Bodine et al., 1982): thus, eccentric contractions, while favoring the increase in Lf without presenting significant changes in PA, may have profound influence on muscle performance (Hoppeler, 2014).

## Molecular and metabolic responses to acute and chronic concentric vs. eccentric loading

Skeletal muscle hypertrophy in response to exercise is the result of the addition of new contractile material, regulated by different molecular mechanisms, involving translational enhancement of muscle protein synthesis (Hoppeler et al., 2011; Atherton and Smith, 2012; Hoppeler, 2016). A small number of studies investigated the molecular mechanisms of muscle hypertrophy in response to concentric vs. eccentric exercise, particularly in humans; the existing work focused on the acute rather than on the chronic molecular responses, and only few investigations reported architectural and morphological adaptations over chronic resistive loading interventions (Blazevich et al., 2007; Franchi et al., 2014, 2015; Timmins et al., 2016a).

For this reason, there is currently no clear consensus as to what mechanisms regulate the structural remodeling of skeletal muscle with ECC vs. CON RT.

This section shall examine the possible molecular mechanisms regulating the responses to ECC and CON loading, from the animal and human data available up to present date, with the objective to better clarify if specific molecular alterations could explain such distinct structural adaptations.

### Muscle protein synthesis after acute and chronic concentric vs. eccentric loading

The distinct remodeling patterns of ECC vs. CON RT observed in regional hypertrophy and muscle architecture point to a contraction-specific muscle growth that may be regulated by distinct whole and regional muscle anabolism. A total of four human investigations were identified and none of them found any notable differences in MPS between the two loading modalities. Phillips et al. (1997) reported similar increase in mixed protein synthesis at 3, 24, and 48 h after single CON and ECC exercise bouts (not matched for work). Likewise, Cuthbertson et al. (2006) found no differences in myofibrillar fractional synthetic rates (FSR) following 12 min step up (CON) or step down (ECC). When the loading paradigms were matched for work, myofibrillar MPS presented greater increase at 4.5 h after ECC RT compared to CON RT, with no differences found after 8.5 h (Moore et al., 2005). We recently explored the potential differences in anabolic response between ECC and CON loading and at different vastus lateralis muscle sites (at the middle of the muscle belly—MID—and at the distal portion, close to the knee myotendinous junction—MTJ; Franchi et al., 2015). The rationale for these measurements being that different architectural adaptations could be governed by contraction-specific responses in MPS; in addition, in a previous study of similar design, distinct patterns of regional hypertrophy were found, as distal growth was associated to ECC RT whereas CON RT seemed to favor increase of mid muscle ACSA (Franchi et al., 2014). Not only ECC and CON (matched for relative maximum load) did not differ in the quantity of myofibrillar FSR (measured over 4 weeks period, using deuterium-oxide tracing technique), but, surprisingly, no differences were observed between muscle sites. Interestingly, Fujita et al. (2007) showed that *de novo* sarcomere assembly in C2C12 myotubes can occur without the requirement for newly synthesized proteins. Collectively, these data suggest that, during muscle structural remodeling, assembly of sarcomeres may be independent of the quantity of new contractile material (i.e., proteins) synthesized. Hence, the different structural adaptations in response to ECC and CON cannot be determined merely through quantifying longer-term MPS responses. In further support of this concept, previous work by Garma et al. (2007) showed that, in rodent muscle, under conditions of equivalent volumes of force integral, both concentric and eccentric loading programmes (i.e., and isometric too) presented near identical activation of processes leading to anabolic response. Similar increase of total mRNA content, IGF-1 mRNA and protocollagen 3 were observed, as well as a decrease in myostatin mRNA. Lastly, in a more recent study on rats, mTORC1 signaling was not modulated by the contraction mode, rather the key factor seemed to be the force-time integral (Ato et al., 2016).

### Exercise-induced muscle damage (EIMD)

Some research suggests that EIMD may play an important role in mediating muscle growth, speculating that the associated inflammatory response and protein turnover could be critical for increase in muscle size (Evans and Cannon, 1991). As EIMD is found predominantly after ECC exercise bouts, but not CON (Fridén et al., 1986; Faulkner and Brooks, 1993), this has prompted researchers to postulate that ECC RT could lead to higher hypertrophic responses: nevertheless, as showed in Section The Contribution of Chronic Concentric vs. Eccentric Loading to Muscle Hypertrophy of the present review, even at high training intensities, many studies cannot clearly affirm which training mode results in greater long-term hypertrophy. Moreover, it is widely known that EIMD decreases since after the first exercise bout (McHugh, 2003; i.e., the repeated bout effect phenomenon) because of several factors, including neural adaptations and structural remodeling of the extra-cellular matrix (ECM; Hyldahl et al., 2017). Thus, the real contribution of EIMD in distinct long-term hypertrophic adaptations to ECC vs. CON RT remains to be determined.

### Satellite cells activity after acute and chronic concentric vs. eccentric loading

If MPS and EIMD do not fully explain the contraction-specific skeletal muscle remodeling patterns, further answers may be found in the satellite cells activity in response to ECC vs. CON RT.

During muscle repair following EIMD and during subsequent hypertrophic adaptations, the role of satellite cells is central for muscle growth (Fry et al., 2017). Satellite cells are stem cell situated between the basal lamina and the sarcolemma of skeletal muscle fibers (Mauro, 1961), which, upon activation, can proliferate, and ultimately fuse with existing fibers, thus leading to an increase in muscle fibers size (Harridge, 2007). It is noteworthy to mention that greater satellite cells activity has been observed after a single bout of ECC exercise (with consequent EIMD showed) compared to load-intensity matched CON (with no damage; Hyldahl et al., 2014). When we consider RT programs overtime, chronic CON RT only has lead to an increase in satellite cells pool, compared to chronic ECC RT (Farup et al., 2014). Thus, these data suggest that distinct contractions can differently modulate satellite cells activity over RT periods.

A recent study highlighted the very close relationship between myogenic progenitor cells (MPCs) and the ECM that surrounds muscle fibers in response to hypertrophic stimuli (Fry et al., 2017). Fry and colleagues showed that when MPCs are activated by mechanical overload, they proliferate and will release exosomes containing different micro RNAs (miRNA) into the ECM-niche. Such cross talk leads to a reduction of collagen mRNA expression in the fibrogenic cell, and thus appears to regulate ECM deposition during muscle structural remodeling. Conversely, the absence of activated satellite cells results in excessive ECM deposition and attenuated myofibre growth (Fry et al., 2017). Furthermore, in a recent investigation, a reduced cohort of satellite cells did not prevent serial sarcomeres addition after chronic stretch in mice soleus, but resulted in significantly altered muscle morphology (reduced fiber CSA with concomitant fibrosis and ECM hypertrophy; Kinney et al., 2017). This proves that satellite cells play a crucial role in controlling ECM and muscle structural remodeling.

### Evidences of enhanced extra-cellular matrix remodeling in response to eccentric contractions

The previous sub-section has pointed out that the cross-talk between mechanical stimuli, satellite cells activation and ECM remodeling could be differently modulated after ECC vs. CON contractions.

A recent human study by Hyldahl et al. (2015) investigated the ECM remodeling contribution to the repeated bout effect, the physiological protective muscular adaptations that usually occurs after a single bout of ECC RT.

The present study presented two major results. Firstly, a global transcriptomic analysis showed that ECM gene networks alteration following an ECC RT bout (compared to the non-exercised leg) were related to early deadhesion (Tenascin C highly activated 2 days post-exercise), force loss and muscle damage, suggesting that these acute responses represented possible source of muscular adaptations in the repeated bout effect process. Furthermore, the ECM collagen genes, both fibrillar (type I and III) and basal lamina (IV) types, showed a prolonged response to one single bout of lengthening contractions, being markedly expressed even after 27 days from the exercise session, suggesting a possible delayed remodeling activity of ECM. In addition, the TGF-β/Smad signaling pathway was found highly activated at the same time point, supporting the above mentioned gene expression data, as TGF-β is regarded as an up-regulator of mechanical loading-derived collagen synthesis (Verrecchia et al., 2001). The present data support the contention that ECM may have a strong role in muscle remodeling and structural assembly after lengthening contractions. However, in this study, no responses to shortening contraction were investigated. Nevertheless, in a previous study on rats (Heinemeier et al., 2007), myostatin mRNA was found decreased the most after ECC compared to CON exercise, together with a larger increase of MGF (Mechano-Growth Factor), TGF-β-1 (transforming Growth Factor β-1, a marker of collagen expression) and CTGF (Connective Tissue Growth Factor) mRNA values in response to ECC training. Collagen type I α1 also presented a larger increase after ECC and Isometric exercise compared to CON; these data suggest once again that extra-cellular matrix-related remodeling could be one of the keys to interpret differences in muscle structural re-assembly in response to lengthening vs. shortening contractions (Mackey and Kjaer, 2016).

### Different gene responses and cellular signaling pathways after acute and chronic concentric vs. eccentric loading

The only study that investigated the gene expression profile of concentric vs. eccentric exercise (unilateral stepping up and down of a small jump box) (Kostek et al., 2007) found that fifty-one genes were differently expressed after 3 h from ECC vs. CON RT. These genes were mostly related to protein turnover, cellular stress and sarcolemmal-structural remodeling. Differences in single genes expression (FBXO32, SIX1, CSRP3 and MUSTN1) were detected between the distinct loading modalities utilized: the four genes targeted, uniquely regulated by lengthening contractions, may play specific roles in skeletal muscle as regulating protein turnover, being potentially responsible for fiber-type change and involved in mechano-transduction processes. Thus, these data seem to support the idea that ECC and CON contractions/training modalities trigger distinct and unique gene networks.

When focusing on skeletal muscle signaling, some interesting findings are worth being reported on the cellular responses to either ECC or CON contractions. Wretman et al. (2001) observed that, in isolated rat muscle, greater increases in phosphorylation of p38 and ERK 1/2 mitogen-activated protein kinases (MAPKs) were induced by ECC compared to CON stimulus. These results were later confirmed by our lab on humans (Franchi et al., 2014), as we observed a greater activation of p38, ERK ½ and p90RSK after 30 min of ECC RT compared to CON. Intriguingly, ERK ½ expression was previously shown to regulate CON vs. ECC growth pathways in cardiomyocytes, suggesting MAPKs are involved in regulating architectural remodeling processes in both cardiac and skeletal muscle (Kehat et al., 2011). Thus, it is interesting to underline that the MAPKs signaling pathways are indeed differently regulated by distinct contractions.

Martineau and Gardiner (2001), observed that MAPKs activation was quantitatively related to tension, with ECC providing the greater stimulus compared to shortening muscle actions. It is well established that mechanical tension is one of the crucial factors influencing muscle growth (Goldberg et al., 1975; Fry, 2004) and also that eccentric contractions are associated with a greater development of tension compared to concentric contractions (Katz, 1939). Interestingly, a successive study, in which the force × time integral (FTI) was manipulated by changing the force of contraction, demonstrated that p38 and FAK phosphorylation correlate with the FTI (Rahnert and Burkholder, 2013) indicating a possible intimate relationship between those targets and the nature of the mechanical stimulus applied on to the muscle. This observation, lead us to investigate the role of FAK activation (at the y-397 tyrosine site) between different contractions (lengthening vs. shortening) and at distinct muscle sites (mid muscle vs. MTJ): a preliminary data analysis showed that y-397FAK activation was greater after a single ECC RT session compared to CON (Franchi et al., 2016b). Moreover, such activation appeared to be muscle site-dependent (MTJ > MID), supporting the regionality/heterogeinity of hypertrophic and structural remodeling adaptations to distinct loading modes.

In conclusion, it appears that when both contraction types are applied to the muscle with matched high intensity and work, similar response in protein synthesis can be observed, with a small potential difference only at very early time points (4.5 h after an exercise bout- Moore et al., 2005). Nonetheless, chronically assessed protein synthetic response (i.e., evaluated within a period of 4 weeks) does not highlight any significant differences between ECC vs. CON (Franchi et al., 2015). Distinct satellite cells activity, ECM-related gene expression and cellular signaling have been observed both in animals and humans, suggesting that ECC may trigger greater connective tissue/structural remodeling. Although, further research is needed to clarify the link between morphological and molecular adaptations, these phenomena could be linked/regulated to/by a greater activation of the MAPK family and mechano-transductors pathways (Wretman et al., 2001; Franchi et al., 2014, 2016b).

## Conclusions

Although, ECC RT has been usually associated to greater increases in muscle mass compared to CON RT, the present review clearly illustrated that the findings presented in the literature are too varied to clearly affirm which training mode leads to greater long-term muscle growth. Furthermore, when both exercises paradigms are matched for either maximum load or work, the hypertrophic responses are very similar. What appears to be different is how this increase in muscle size is reached, as distinct contraction-specific adaptations in muscle architecture are found. In addition, different molecular and myogenic mechanisms have found distinctly activated after ECC vs. CON exercise bouts, suggesting that these responses could be underlying the structural remodeling patterns previously described: further investigations are needed to establish the strength of such potential micro-to-macro connections. At present time, the aforementioned scenarios are clearly described for a young population, whereas this is yet to be fully elucidated in older individuals and in clinical settings.

## Author contributions

MF, NR, and MN contributed altogether to the conception and writing up process of this manuscript.

### Conflict of interest statement

The authors declare that the research was conducted in the absence of any commercial or financial relationships that could be construed as a potential conflict of interest.
